# The culturable endophytic fungal communities of switchgrass grown on a coal-mining site and their effects on plant growth

**DOI:** 10.1371/journal.pone.0198994

**Published:** 2018-06-14

**Authors:** Ye Xia, Amna Amna, Stephen Obol Opiyo

**Affiliations:** 1 Department of Plant Pathology, The Ohio State University, Columbus, OH, United States of America; 2 Department of Plant Sciences, Quaid-i-Azam University, Islamabad, Pakistan; Estacion Experimental del Zaidin, SPAIN

## Abstract

Plants have a diverse endophytic microbiome that is functionally important for their growth, development, and health. In this study, the diversity and specificity of culturable endophytic fungal communities were explored in one of the most important biofuel crops, switchgrass plants (*Panicum virgatum* L.), which have been cultivated on a reclaimed coal-mining site for more than 20 years. The endophytic fungi were isolated from the surface-sterilized shoot (leaf and stem), root, and seed tissues of switchgrass plants and then cultured for identification. A total of 1339 fungal isolates were found and 22 operational taxonomic units (OTUs) were sequence identified by internal transcribed spacer (ITS) primers and grouped into 7 orders and 4 classes. Although a diverse range of endophytic fungi associated with switchgrass were documented, the most abundant class, order, and species were Sordariomycetes, Hypocreales, and *Fusarium spp*. respectively. About 86% of the isolated endophytic fungi were able to enhance the heights of the shoots; 69% could increase the shoot fresh weights; and 62% could improve the shoot dry weights after being reintroduced back into the switchgrass plants, which illustrated their functional importance. Through the Shannon Diversity Index analysis, we observed a gradation of species diversity, with shoots and roots having the similar values and seeds having a lesser value. It was observed that the switchgrass plants showing better growth performance displayed higher endophytic fungal species diversity and abundance. It was also discovered that the rhizosphere soil organic matter content was positively correlated with the fungal species diversity. All these data demonstrate the functional association of these beneficial endophytic fungi with switchgrass and their great potential in improving the switchgrass growth and biomass to benefit the biofuel industry by reducing chemical inputs and burden to the environment.

## Introduction

The global demand for energy is expected to increase by more than 50% by the year 2025 [1]. The increasing problems and concerns regarding greenhouse gas emission due to burning of the fossil fuels and the need for rural economic development are key factors stimulating the development of national and international strategies to increase sustainable biofuel crops production for bioenergy generation.

Feedstocks, such as Switchgrass (*Panicum virgatum L*.) and *Miscanthus sinensis*, are being developed as sources of biofuels in order to meet the ever-increasing energy needs in an environment friendly way. Switchgrass is a perennial warm-season C-4 grass native to North America. Due to its wide geographic distributions and adaptation to diverse edaphic conditions, switchgrass is considered as one of the most important bioenergy crops in developing the sustainable bioenergy [2]. In addition, switchgrass can be cultivated on the marginal or abandoned agricultural lands, such as coal-mining sites [3], thereby reducing the competition with food crops for lands and other resources. Switchgrass also displays efficient water utilization owing to its deep perennial root system, which also prevents soil erosion and helps in carbon sequestration [4]. Therefore, this cellulosic crop could potentially be a critical component of an economically profitable and environmentally friendly biofuel industry in North America and possibly other countries [2].

Plant-associated microbiome is defined as the microbial community that plays critical roles in plant growth, development, and resistance to diverse stress conditions [5]. These microbes can exist in different plant tissues, including the stems, leaves, seeds, and roots. Fossil evidence indicated that interactions between these microbes and plants originated about 400 million years ago [6]. The classical definition of plant endophytes is: “All organisms inhabiting plant organs that at some time in their life, can colonize internal plant tissues without causing apparent harm to the host” [7]. These endophytes may have closer relationships with the host plants than the other plant-associated microbes, such as the rhizosphere or phyllosphere microbes, since they need to manipulate the plant immunity to enter and develop in the host plants. Optimization of the endophytic elements of the plants has demonstrated great potential in improving agricultural productivity in terms of water and nutrient uptake and utilization by the plants [8], detoxification of the environmental chemicals [9] [10], and improvement of the plant resistance to biotic and abiotic stress [11] [12].

Around 300,000 ha of abandoned coal-mining sites exist in Kentucky, USA [3], and all are in need of urgent services for improvement of the ecosystem. The switchgrass and other warm season grasses were cultivated on the western Kentucky coal-mining sties for ecological maintenance more than 20 years ago [3]. Switchgrass production could be improved by the use of conventional fertilizers and pesticides, which may increase the production and environmental costs. On the other hand, identification of beneficial endophytic microbes of switchgrass would enable their better utilization thereby making switchgrass production sustainable and efficient.

The development of next generation sequencing and multiple omics techniques, such as metagenomics and metatranscriptomics approaches have increased our knowledge about the microbial community composition residing within the host plants and their associated functions [13]. For example, the plant genotypes and defense hormone salicylic acid pathway have been shown to play critical roles in recruiting their associated endophytic microbiome for plant development and growth [14] [15] [16] [17] [18]. However, the detailed mechanisms of such functional association of plant-microbiome and the environmental factors affecting them are yet to be fully understood. Current approach to characterize and study the plant-associated microbial community composition is by using both culture-dependent and culture-independent methods [19]. The culture-dependent techniques might have limitations to capture large amounts of microbes that cannot be cultured on media with known methods [20]. However, it also has its advantages, since diverse microbial endophytes could be isolated, identified, and further characterized for their function on plants separately. A synthetic beneficial endophytic microbial community could also be created from the isolated beneficial endophytes for the benefit of host plants. Although recent research has shown the great potential of endophytes in promoting plant growth and biomass [21] [22] [23] [24], further understanding of the related mechanisms and more efficient utilization of these endophytes could be very important for the sustainable bioenergy production of switchgrass and other biofuel crops to meet bioenergy requirements.

In this study, we aimed to investigate the culturable endophytic fungal community associated with the switchgrass grown as a ground cover for ecological maintenance on a coal-mining site for more than 20 years in Kentucky, USA [3] [19]. Herein, a highly degraded coal-mining site was identified and divided into two parts-part 1 and part 2. The switchgrass plants displayed more vigorous growth on part 1 than part 2 (S1 Fig). Therefore, we hypothesized that the increased vigorous growth of the switchgrass on part 1 may be due to the beneficial microbial associations, and these microbes may help the switchgrass with their establishment, growth, and development. Previously we had investigated the culturable endophytic bacterial community of switchgrass plants and their effects, most of which had shown activity in enhancing the growth of switchgrass [19]. Our overarching objective was to identify the culturable endophytic fungi associated with the switchgrass, which can be utilized for improvement of plant growth, development, and biomass; and to study the possible effects of different soil conditions on the endophytic fungal community structures and functions, which might also contribute to the switchgrass performances.

## Materials and methods

### Switchgrass plants and soil sample collections from one reclaimed coal-mining site

The switchgrass plants were collected in July 2011 from one reclaimed coal-mining site in western Kentucky, where they were established as a monoculture during reclamation approximately 20 years ago [3]. The selection site was located between the GPS coordinates of W:087 25’ 04,13” to 087 25’ 07, 31” longitude and N:037, 15’ 46, 21” to 037, 15’ 50, 60” latitude. The site was grouped into two parts based on the plant performance. The plants on part 1 showed better vigorous growth compared with those on part 2 (S1 Fig). About 40 representative switchgrass plants grown from each part were collected and grouped for 5 replicates. The representative plant rhizosphere soil attached to the roots of switchgrass plants (0–2 cm around the roots) from both parts was also collected and grouped for 3 replicates. The soil was further analyzed for pH, organic matter, and other major nutrient contents by using the method mentioned in Soil & Plant Analysis Council, 2000 [25]. The same amount of dry rhizosphere soil that passed through a 10-mesh (2-mm opening) sieve was mixed with water in 1:2 ratio (vol/vol) and the pH was evaluated with a pH meter. The remaining solution was mixed with different buffers for extraction of different elements and their analysis. For example, the determination of N, P, K, and the organic matter contents were determined by using the Mehlich buffer solution 1 (0.05 N hydrochloric acid and 0.025 N sulfuric acid) [25] [26].

### Isolation of fungal endophytes from within shoot, root, and seed tissues of the switchgrass plants

Shoot segments, about 3–5 cm, having equal weights and comprising of leaves and stems, and also the root tissues, were cut from the whole switchgrass plants. The same amount seeds were also separated from the collected plants. All these plant samples from different tissues were washed with tap water to remove the attached soil and other debris. Following this, the plant segments and seeds were rinsed with 95% ethanol for 2 minutes, then immersed in 30% Clorox bleach for around 15 minutes to destroy the surface microbes and their DNA, and rinsed with sterile water 3–5 times as mentioned in a previous reported method [27, 28]. The shoot and root samples were then cut into 1–1.5 cm length pieces, and the seeds were cut into halves. Around 6 pieces of each plant tissue were put on one PDA media plate (Potato Dextrose Agar, Sigma-Aldrich, USA). This was done with all the different plant tissues. The plates were incubated in an incubator with a constant temperature of 26°C for 5–7 days. The numbers of the emerged fungal isolates from different tissues of each plant were recorded separately, isolated, and further purified by growing on the PDA plates for 2–3 times [27] [29].

### DNA extraction, ITS rDNA amplification, sequencing, and fungal species identification

The isolated fungi were grouped based on their morphological features and the representative ones were further identified by DNA sequencing. The representative and purified individual colonies were grown on the PDA medium plates and kept at 26°C in an incubator for about 5 days. The fungal DNA was then extracted by using a Zymo Research fungal/bacterial DNA miniprep kit (Zymo Research, USA) following the manufacturer’s instruction. The ITS (Internal Transcribe Spacer) has a higher degree of variation than most of the other genic regions of rDNA [30]. Therefore, it was selected for the related sequencing in this study. The ITS rDNA amplification was performed in a 50 μl reaction containing 5 μl DNA template (1-20ng), 3 μl of *Taq* buffer and 1 U *Taq* DNA polymerase, 5 μl of 10 μM primers ITS1 (5’- tccgtaggtgaacctgcgg -3’) and ITS4 (5’- tcctccgcttattgatatgc-3’), 3mM Mgcl_2,_ and 3 mM dNTPs (Fermentas Inc., USA). The PCR amplification was performed on an icycler PCR machine (Bio-Rad Laboratories, CA): first with an initial denaturation (94°C for 5 min), then 50 cycles of amplification (94°C for 1 min, 54°C for 1 min, 72°C for 1.5 min), and a final extension step (72°C for 5 min). The PCR products were purified using the Zymo DNA (PCR) purification kit (Zymo Research, USA) and quantified with a nanodrop spectrophotometer and sent to the Ohio State University eRAMP center for sequencing. The verified sequences were blasted through the BLASTn searches in the NCBI (National Center for Biotechnology Information database), and the taxonomic resolutions were identified based on the top hits from the database[19].

### Statistical data analysis

The fungal isolates were also grouped into different taxonomic levels (species, order, and class), so that the frequencies and diversity could be evaluated according to their tissue and location distributions (part 1 & 2). All the related statistical analyses were carried out using R software (R Core Team (2017)) [31]. The species diversity was calculated using the Shannon Diversity Index indices [32] and vegan R package developed by Oksanen et al. [33]. Fungal species abundance and diversity for different tissues and locations (part 1 and part 2) were compared using the Hutcheson method [32]. Analysis of Variance (ANOVA) was used to compare fungal species abundance among tissues. And Student’s t-test was used to compare levels of pH, organic matters, and major nutrient contents of rhizosphere soil between two locations.

### Screening for endophytic fungal isolates with plant growth promotion activity

The switchgrass cv. Alamo seeds were washed with 70% ethanol for about 1–2 minutes and washed with a solution of 30% bleach for 15 minutes. The seeds were then rinsed with sterilized water 3–5 times and subsequently stored at 4°C for 24 hours. Individual disks with a diameter of around 0.6cm of these endophytic fungi were put in the center of the PDA medium plates and grown in an incubator maintained at 26°C for 5 days. Further, the fungal spores and mycelia without containing the PDA media were transferred to sterile flasks and the volume were made up to 100 ml with sterilized water. Two sterilized switchgrass seeds were added to each pot filled with Pro-Mix (Premier Horticulture, Ltd, PA, USA) used as potting media, and the mixed 100ml water containing the fungal spores and mycelia was applied to each pot, and 100 ml pure water treatment was used as controls in separate pots with two switchgrass seeds. Each fungal and water treatments comprised of at least 3 pots with 6 plants as replicates. The pots were kept in a green house at a constant temperature of 28°C and 16 hours of light followed by 8 hours of darkness. The shoot growth (aerial heights) as well as the shoot fresh and dry weights (dried at 70°C in an oven for 3 days) of the switchgrass plants with different treatment was recorded at 8 weeks. The experiments were repeated for 3 times and all the data were pooled together for further analysis. The fungi that had the ability in enhancing the switchgrass plant growth and biomass were further identified.

## Results

### The endophytic fungal community diversity and abundance

**Fig 1:**
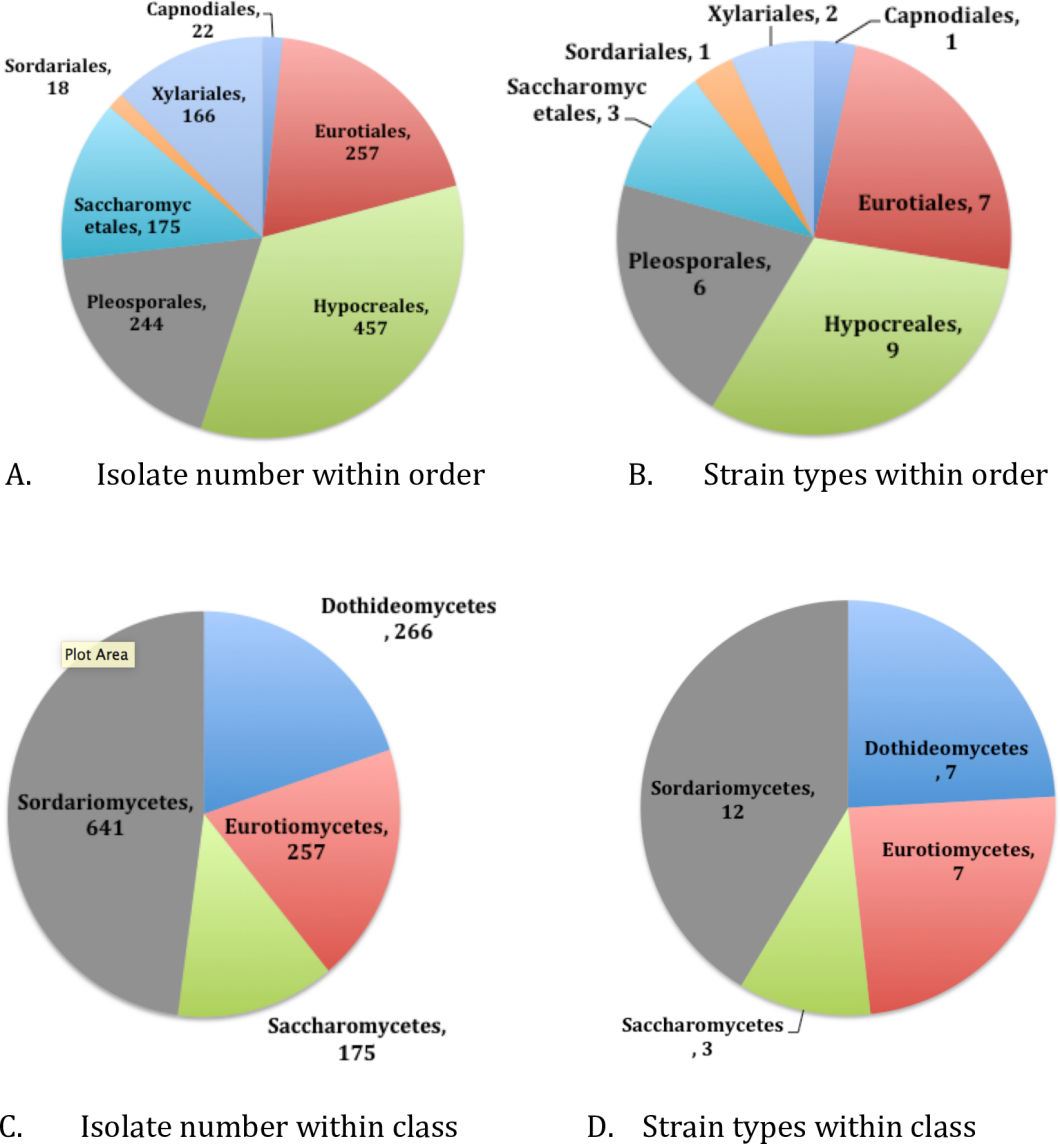
The distribution of endophytic fungal isolates from switchgrass. The isolate numbers (A) and strain types (B) at the order level; and the isolate numbers (C) and strain types (D) at the class level.

A total of 1339 isolates were obtained from the switchgrass plants collected from the coal-mining site. These fungi were first cultured on the PDA media and then sub-cultured two times to get pure individual fungal isolates. Through ITS sequence identification, we demonstrated that these endophytic fungi were classified into 22 OTUs (Operational Taxonomic Units) at species level, which belonged to 29 different strains (top hits in NCBI). Further classification indicated that the 22 species belonged to 7 orders and 4 classes (Fig 1A–1D). The distributions of the isolate numbers and strain types of these 7 orders and 4 classes are shown in Fig 1. The order Hypocreales was the most abundant among the isolated endophytic fungi, which included 7 OTUs and 457 isolate numbers constituting approximately 32% and 34% of both the total OTUs and isolate numbers respectively. The order of Hypocreales included mainly the species of *Fusarium spp*. and *Trichoderma spp*. The other fungal orders that had been identified included Saccharomycetales, Capnodiales, Eurotiales, Pleosporales, Sordariales, and Xylariales (Fig 1A and 1B and S1 Table).

**Fig 2:**
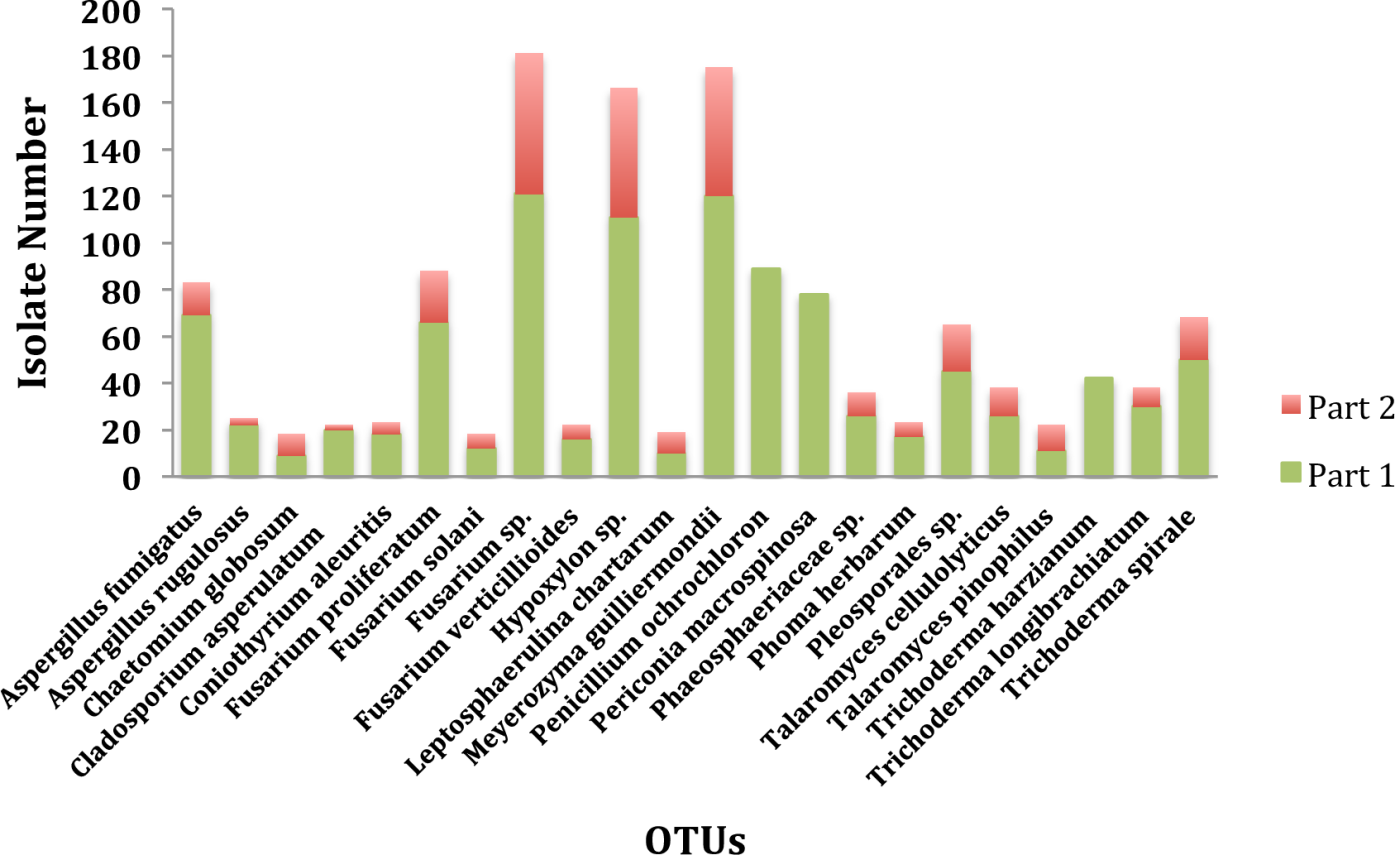
The distribution of endophytic fungi at species level between two locations on coal-mining site.

Among these isolated endophytic fungi, the second abundant order was Eurotiales, comprising 19% of the total isolate number, which included the species of *Aspergillus fumigatus*, *Aspergillus rugulosus*, *Penicillium ochrochloron*, *Talaromyces cellulolyticus*, and *Talaromyces pinophilus*. The third abundant order was Pleosporales, comprising about 18% of the total isolate numbers, which included the species of *Coniothyrium aleuritis*, *Leptosphaerulina chartarum*, *Phaeosphaeriaceae spp*., *Phoma herbarum*, *Periconia macrospinosa*, and *Pleosporales spp*. (S1 Table). The orders of Saccharomycetales and Xylariales both comprised of around 12% of the total isolate numbers. The Saccharomycetales mainly included the species of *Meyerozyma guilliermondii*. The order Xylariales included only *Hypoxylon spp*. Further examination of the distribution pattern of the endophytic fungal isolates showed that the most abundant top 5 species out of a total of 22 species were *Fusarium spp*., *Meyerozyma guilliermondii*, *Hypoxylon spp*., *Penicillium ochrochloron*, and *Fusarium proliferatum* in the descending order. These 5 species comprised approximately 52% of the 1339 total isolates (Fig 2, S1 Table). At the class level, the endophytic fungi could be grouped into 4 classes. The classes with the most abundant isolate numbers (IN) and strain types (ST) were Sordariomycetes (IN: 641, ST:12) followed by Dothideomycetes (IN:266, ST:7), Eurotiomycetes (IN:257, ST:7), and Saccharomycetes (IN:175, ST:3) (Fig 1C and 1D, S1 Table)

### The fungal community diversity associated with specific locations and tissues

Our data showed that out of the total 1339 isolates, 1008 were obtained from the switchgrass plants grown on part 1 (having better vigorous growth), and the remaining 331 were isolated from plants grown on part 2 (having poor growth) (S1 Fig). Based on Hutcheson statistical analysis, it was found that the species abundance of the endophytic fungi in switchgrass plants was significantly higher on part 1 compared to part 2 (Table 1). Three among 22 OTUs, namely *Penicillium ochrochloron*, *Periconia macrospinosa*, *and Trichoderma harzianum*, were not present in plants collected from part 2. All the other species were isolated from plants collected from both parts (Fig 2). The data for Shannon Diversity Index analysis method revealed that the endophytic fungal community species diversity from the two parts significantly differed between the plants from two parts and had a greater value for isolates from plants in part 1 (Hs = 2.83) than those from part 2 (Hs = 2.06) (P<0.05, Table 2).

**Table 1:** The abundance of the endophytic fungi from switchgrass.

Relative abundance			
Number (n)	Part 1	Part 2	p-value
**Total (Part 1 = 1008, Part 2 = 331)**	**0.75**	**0.25**	**<0.001**
**Shoot (Part 1 = 535, Part 2 = 171)**	**0.76**	**0.24**	**<0.001**
**Root (Part 1 = 356, Part 2 = 121)**	**0.81**	**0.19**	**<0.001**
**Seed (Part 1 = 117, Part 2 = 39)**	**0.77**	**0.23**	**<0.001**

The fungi were collected from different tissues of switchgrass grown from different locations (part 1 or part 2) on a coal-mining site. The p-values were calculated using the function cor.test of R statistical software.

**Table 2:** Shannon indices values and difference analysis of endophytic fungal species diversity.

Plant	Factors	Shannon indices	p-value
**Total**	**Part 1 vs. Part 2**	**2.83 vs 2.06**	**<0.001**
**Seed**	**Part 1 vs. Part 2**	**2.59 vs 1.54**	**<0.001**
**Shoot**	**Part 1 vs. Part 2**	**2.98 vs 2.38**	**<0.001**
**Root**	**Part 1 vs. Part 2**	**2.94 vs 2.25**	**<0.0001**
**All**	Shoot vs. Root	2.68 vs. 2.60	0.17
**All**	**Shoot vs. Seed**	**2.68 vs. 2.05**	**<0.001**
**All**	**Root vs. Seed**	**2.60 vs. 2.05**	**<0.001**

The comparison was analyzed between two locations (Part 1 or Part 2) and among different plant tissues of switchgrass plants from the coal-mining site with the p-values being calculated by Hutcheson t-test.

**Fig 3:**
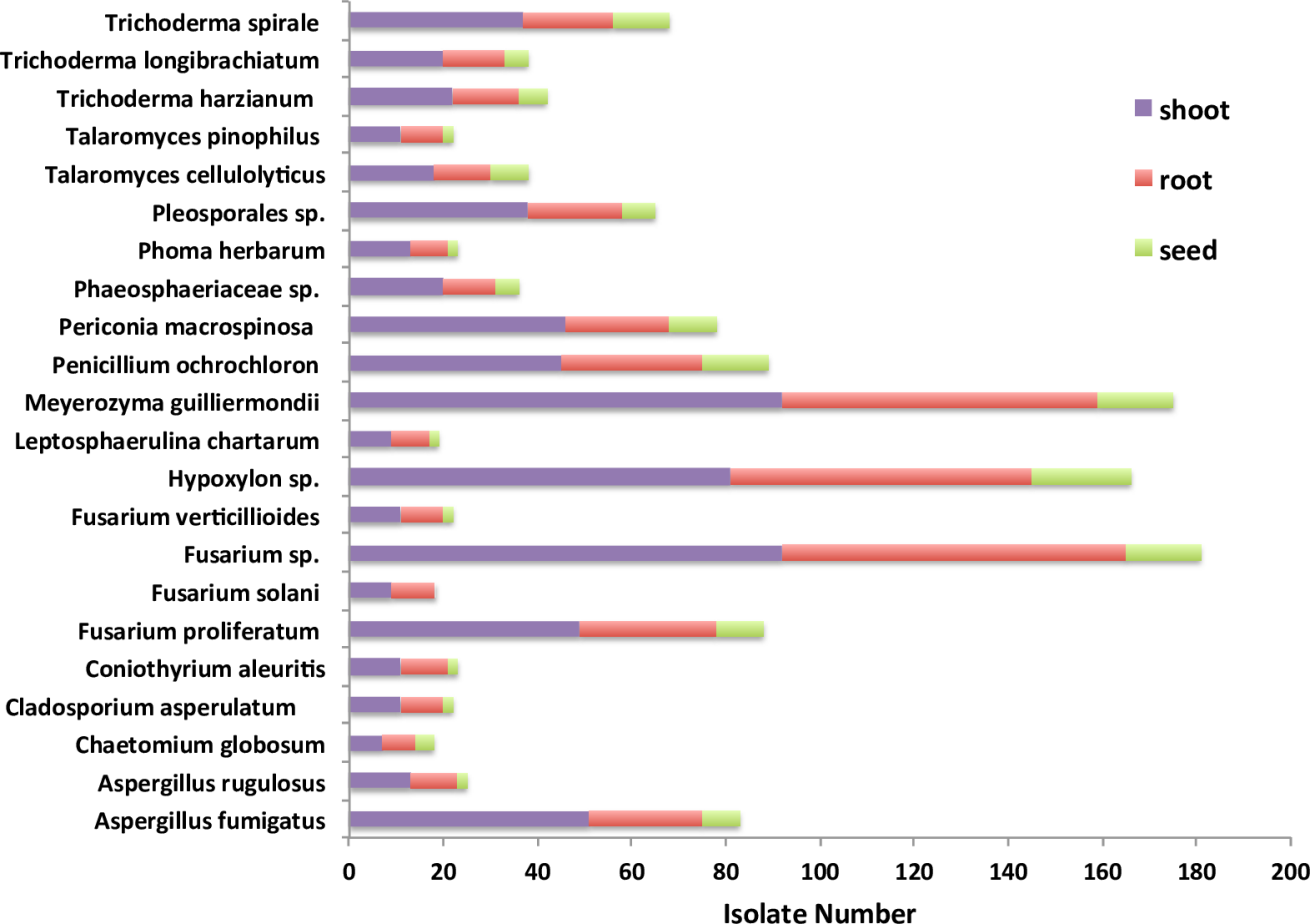
The distribution of endophytic fungi at species level. The data were analyzed among different tissues of switchgrass grown on the coal-mining site.

The species diversity and abundance of the endophytic fungi among different tissues was also compared (Tables 2 and 3). Of these 1339 isolates, the shoot community had 706 isolates that belonged to 22 OTUs; the root community had 477 isolates that belonged to 22 OTUs; and the seed community had 156 isolates that belonged to 21 OTUs (Fig 3). The fungal species abundance was highest in shoot tissues, followed by root and seed tissues from plants in both parts (Table 3). Out of all the 22 species, 21 species were widely distributed in all tissues, but *Fusarium solani* was not found in seed tissue. The most abundant species in all the tissues were *Fusarium spp*. followed by *Meyerozyma guilliermondii* and *Hypoxylon spp*. The species diversity was similar in shoot (Hs = 2.68) and root (Hs = 2.60) tissues while the seed tissues exhibited a lesser species diversity (Hs = 2.05)(Table 2).

**Table 3:** Endophytic fungal species abundance among tissues.

	Relative abundance	Relative abundance
Tissue Type	Part 1 (P-value <0.001)	Part 2 (P-value < 0.001)
**Shoot**	0.58	0.53
**Root**	0.32	0.36
**Seed**	0.10	0.11
		

The analysis was based on the tissue distribution of plants collected from each location (Part 1 vs. Part 2) with the p-values being calculated by ANOVA (Analysis of Variance).

### The plant rhizosphere soil nutrient content analysis and its correlation with the endophytic fungal species diversity

All the switchgrass plants were established at the same time on this coal-mining site, and the environmental conditions, such as the temperature and rainfall, were fairly similar. To further explore the probable factors responsible for the differences in fungal species diversity and abundance, the same amount of dry rhizosphere soil (0–2 cm around the plant roots) was further analyzed. The results indicated that the soil pH, organic matter, and nitrogen contents of plant rhizosphere soil obtained from part 1 were significantly higher than those from part 2. However, the contents of P and K were significantly lower from part 1 than those from part 2 (Table 4). Thus, there existed a difference in composition of soil collected from the two parts of the coal-mining site.

**Table 4:** The levels of pH, organic matter, and major nutrient contents of plant rhizosphere soil.

	Part 1	Part 2	P value
**soil organic matter (%)**	10.50±5.31	1.71±0.34	<0.05
**soil pH**	7.48±0.25	6.08±0.23	<0.05
**N (%)**	0.16±0.003	0.04±0.002	<0.05
**P (mg/kg)**	0.67±0.14	1.66±0.63	<0.05
**K (mg/kg)**	52.33±12.86	70.33±3.17	<0.05

The data were analyzed between two locations (part 1 and part 2) on the coal-mining site and the p-values were calculated by Student’s t-test.

Further, the correlation between rhizosphere soil nutrient contents and the endophytic fungal species diversity was analyzed. It was found that only the soil organic matter content had high positive correlation with the endophytic fungal species diversity in both parts, but not with the other factors, such as the pH, N, P, and K contents (Table 5).

**Table 5:** The correlation between Shannon Index value and soil variables.

	Shannon Index Part 1		Shannon Index Part 2	
	Correlation	P-value	Correlation	P-value
**Soil organic matter (%)**	0.98	<0.001	0.66	<0.05
**Soil pH**	-0.01	0.987	0.58	0.061
**N (%)**	0.39	0.739	0.50	0.085
**P (mg/kg)**	-0.06	0.962	0.99	<0.001
**K (mg/kg)**	-0.84	<0.001	-0.27	0.674
				

The p-values were calculated using the function cor.test of R statistical software.

### The screening for endophytic fungi with potential to enhance switchgrass growth and biomass

**Fig 4:**
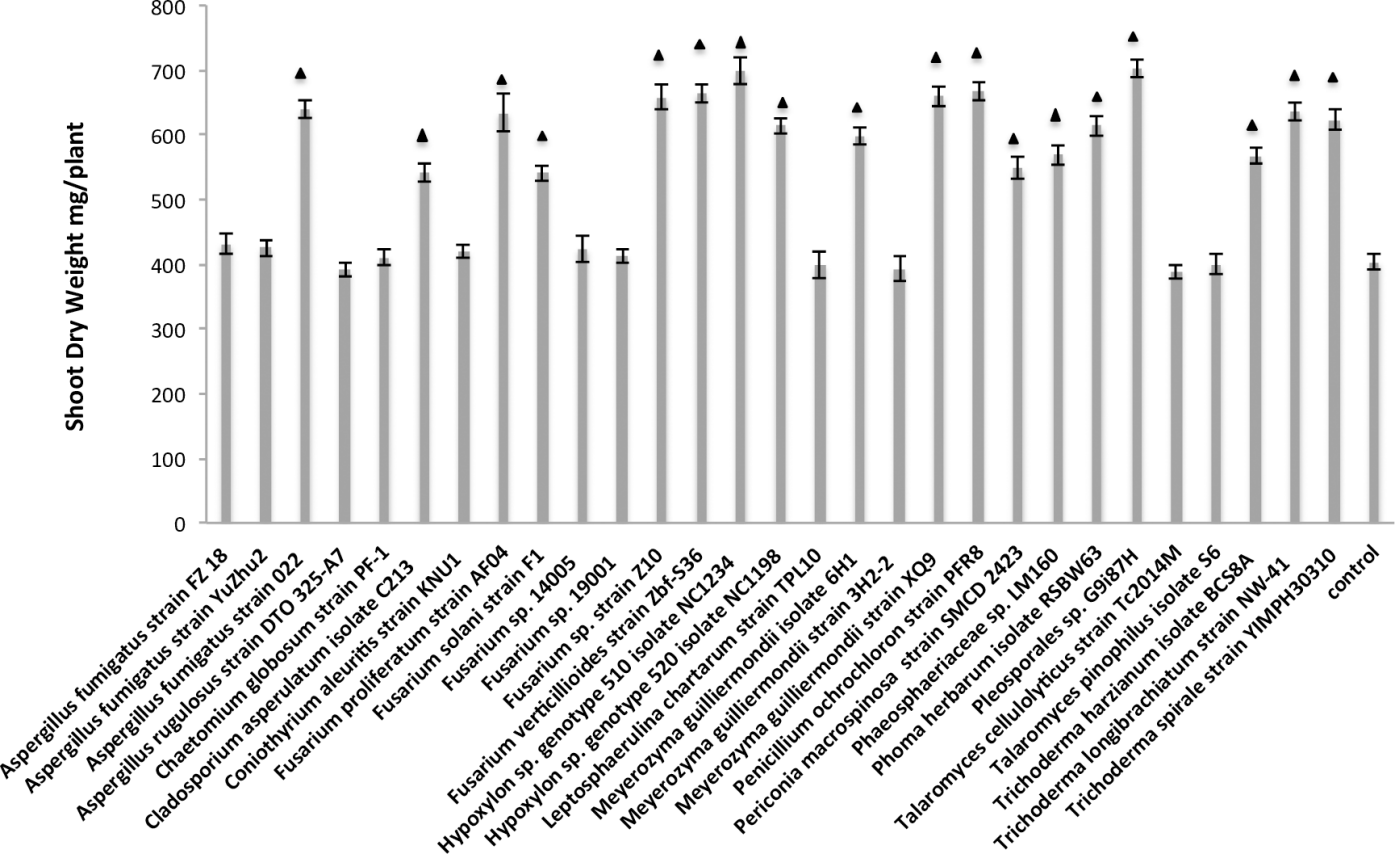
The shoot dry weights of the switchgrass plants. The plants were treated with water control and the water broth containing diverse fungal spores and mycelia at 8 weeks under greenhouse condition. The triangle represents the significant difference existing between the fungal broth treated and water treated plants; the data were further analyzed by the Student’s t-test (P<0.05).

As mentioned above, we isolated a total of 29 strains that could be grouped into 22 different fungal species and 4 classes. We tested all of them for their potential to enhance the switchgrass growth and biomass by inoculating the surface sterilized switchgrass seeds through soil drench with the water containing the fungal spores and mycelium. Out of 29 different fungal strains inoculated on the switchgrass plants, approximately 86% were capable of significantly increasing the heights of the plant shoots (S2 and S3 Tables). The top 5 strains that enhanced the switchgrass plant height were *Pleosporales sp*. *G9i87H*, *Hypoxylon sp*. *genotype 510 isolate NC1234*, *Fusarium sp*. *strain Z10*, *Fusarium verticillioides strain Zbf-S36*, and *Meyerozyma guilliermondii strain XQ9* (S2 and S3 Tables). We also found 20 (69%) and 18 (62%) strains to significantly increase the switchgrass shoot fresh (S2 Fig) and dry weights (Fig 4), respectively.

## Discussion

The cellulosic crop, switchgrass (*Panicum virgatum L*.*)* is a large and important component of an economically profitable and environmentally friendly biofuel industry of North America [34].The identification and application of endophytic fungi possessing growth and biomass promoting activities and imparting resistance to biotic and abiotic stresses could be critical for its sustainable production. Till date, some studies have explored the diversity and function of endophytic fungal communities of the switchgrass [27] [35] [36]. However, the endophytic fungal community compositions and related functions associated with the switchgrass grown in some special conditions, such as on coal-mining sites, have not been widely studied.

One of the primary aims of this study was to examine the culturable members of the endophytic fungal community of switchgrass from a coal-mining site and their potential beneficial effects on the growth and biomass of switchgrass plants. Our findings in this study revealed a broad range of endophytic fungi, which could be classified into 4 classes, 7 orders, 22 species, and 29 strains. It was noted that Hypocreales was the most abundant order, which included mainly the species of *Fusarium spp*. and *Trichoderma spp*. This could be due to the ability of these fungi to enhance the plant growth and resistance to diverse stress conditions that has been widely reported. For example, *Fusarium verticillioides* has been reported to reduce disease severity caused by the fungal pathogen *Ustilago maydis* on maize [37]. Some strains of the *Fusarium spp*. have been reported to enhance the growth of spinach and tobacco plants [38] [39]. Several strains of *Trichoderma* have shown the bio-control effects against *Pythium ultimum* growth in pea plants, and they could also promote the pea plant growth significantly. In addition, these strains could reduce the increased activities of the enzymes of the C, N, and P cycles caused by the *Pythium* infection, thereby decreasing plant damage and C leakage [40].

Ghimire et al. [27] identified a wide range of fungal species representing 18 taxonomic orders in switchgrass grown in the native prairies in Oklahoma, U.S.A, suggesting the presence of a high level of diversity of the endophytic fungal community in switchgrass plants in that area. In their study, the order Hypocreales and the genera *Alternaria*, *Codinaeopsis*, *Fusarium*, *Gibberella*, *Hypocrea*, *Periconia* were the most commonly isolated endophytic fungi associated with switchgrass. They also reported that the *Ectomycorrhizal* fungus *Sebacina vermifera* could enhance the biomass of switchgrass [41]. Klecaewski et al. analyzed the samples of the switchgrass collected from the Midwest of USA and found some of the endophytic fungi had potential to enhance the switchgrass plant productivity [35]. For example, they found that *Phaeosphaeria pontiformis*, *Epicoccum nigrum*, *Colletotrichum sp*., *Alternaria sp*. could significantly increase the total biomass in shoot tissues of switchgrass by 25–33%. In their study, the most commonly detected endophytic fungal genera were *Alternaria*, *Stagonospora*, *Phoma and Epicoccum*, *a*nd the most abundant order was Pleosporales. In our study, the most abundant endophytic fungal order was Hypocreales and *Fusarium*, *Hypoxylon*, *and Meyerozyma* constituted the most abundant genera. The significant difference in endophytic fungi associated with switchgrass plants among different studies could be attributed to different growth and environmental conditions. In our study, the coal-mining site had special soil conditions that may be different from the other lands. Therefore, *Fusarium*, *Hypoxylon*, *and Meyerozyma* may have the ability to survive and develop better than the other fungi under stress conditions, such as those on the coal-mining site, to establish a closer association with the switchgrass plants and benefit their growth and development.

Our study also indicated that the community structure of these culturable endophytic fungi colonizing the switchgrass plants differed among different tissues. A greater number of fungal species were isolated from the shoots than from the roots and seeds (Fig 3). The shoot and root tissues had the similar species diversity based on the Shannon Index values, while the seeds exhibited a lesser species diversity (Table 2). The transmission of fungi from soil and rhizosphere to the above ground parts of switchgrass plants could feasibly be a reason for the difference of abundance and species diversity of these endophytic fungi among different tissues. Some soil and rhizosphere microbes have the potential to be endophytes depending on plant growth stage and environmental conditions [7].

Our data also showed that out of the total 1339 isolates, 1008 were from the switchgrass plants on part 1 with better vigorous growth, and 331 were from those with poor growth performance on part 2 (S1 Fig). Three species, *Penicillium ochrochloron*, *Periconia macrospinos*, and *Trichoderma harzianum*, were only isolated from plants on part 1, but not from those on part 2. And these three species have been reported to have beneficial effects on improving growth and resistance to stress conditions in diverse plants [42] [43] [44].

The endophytic fungal species diversity was significantly higher in switchgrass plants on part 1 than that on part 2 (Table 2). Also, the switchgrass rhizosphere soil on part 1 had significantly higher pH, organic matter, and nitrogen contents, but significantly lower levels of P and K contents compared to those from part 2 (Table 4). The R statistical software analysis indicated that only the plant rhizosphere soil organic matter content has a positive correlation with the endophytic fungal species diversity in the rhizosphere soil from both parts, but not the other nutrient content or pH (Table 5). It has been reported that the soil type and plant genotype may contribute to the plant associated endophytic community composition and function [14] [45]. The pH of the soil has been reported to impact the soil- and plant- associated microbial species diversity in the communities [45] [46]. In our study, we demonstrate the positive correlation of soil organic matter with the endophytic fungal species diversity associated with the switchgrass plants. Our result indicated that the fertile soil with higher organic matter from part 1 might benefit the switchgrass plants and their associated endophytic fungal microbes and promote their interactions. The abundant and diverse endophytic fungi could potentially help the plants grow better and have higher yields and therefore the switchgrass plants may have demonstrated vigorous growth.

Herein, we tested the plant growth promoting activities of the 29 isolated endophytic strains and approximately 86% of these fungal strains significantly increased the switchgrass shoot growth compared with the water controls (P<0.05 Student’s t-test) (S2 Table), and around 60% of them could enhance the switchgrass shoot biomass including dry and fresh weights under greenhouse condition as shown above (Fig 4; S2 Fig). Sixteen strains had the activity in improving both the switchgrass growth and biomass, and the top three were *Pleosporales sp*. *G9i87H*, *Hypoxylon sp*. *genotype 510 isolate NC1234*, and *Fusarium sp*. *strain Z10*.

Future studies using high throughput sequence identification could reveal more information about the obligate endophytic fungi and the improved method to culture more diverse endophytic fungi may help identify more switchgrass associated endophytic fungi and ensure better understanding of the transition of soil or rhizosphere associated fungi into the plants as endophytes. The study of the mechanisms on how these endophytic fungi enhance the plant growth and yield will facilitate their optimum applications. The potential activities and mechanisms of these beneficial endophytic fungi in enhancing plant resistance to biotic and abiotic stresses warrant further investigation. In addition, it would be worthwhile to create a synthetic microbial community to investigate interactions of these fungal microbes in affecting the plant growth and health. All these related studies would facilitate the understanding of basic mechanisms and the applications of these endophytic fungi in enhancing the production of switchgrass as an important biofuel crop and possibly many other crops through a sustainable approach so as to reduce chemical usage and benefit the environment.

## Conclusions

We examined the association between switchgrass plants and their complex endophytic fungal communities grown on a coal-mining site. Our data showed that a majority of the isolated culturable endophytic fungi from the switchgrass were capable of enhancing the switchgrass shoot growth and biomass under greenhouse conditions. Our data also indicated that there might be a high positive correlation of the rhizosphere soil organic matter with the endophytic fungal species diversity and vigorous growth of the switchgrass plants. It was further indicated that there might be functional associations of the endophytic fungi with the switchgrass plants, which may be enhanced by better soil conditions, such as presence of higher organic matter in the rhizosphere soil from part 1 versus part 2. This study highlights the great potential of these fungal endophytes in enhancing agro-ecosystem robustness in this forage and emerging bioenergy crop and possibly many other crops, by way of increased plant growth and yield, and benefit to the environment.

## Supporting information

S1 Fig The growth performance of switchgrass on a coal-mining site. (A) Swtichgrass grown on part1 with better vigorous growth. (B) Swtichgrass grown on part 2 with poor growth performance.
(PDF)

S2 Fig The shoot fresh biomass of switchgrass plants. The switchgrass plants were treated with water control and the water broth containing diverse fungal spores and mycelia at 8 weeks under greenhouse condition. The triangle represents the significant difference existing between the fungal broth treated and water treated plants and the data were further analyzed by the Student t-test (P<0.05).
(PDF)

S1 Table The distribution of OTUs and isolate number at species, order, and class level. (PDF)

S2 Table The effects of 29 endophytic fungal strains (NCBI top hit) on the switchgrass shoot heights. The switchgrass plants were grown under greenhouse conditions.
(PDF)

S3 Table ITs sequences and NCBI accession number of the fungal strains listed in S2 Table. (PDF)

## Abbreviations

PDA: Potato Dextrose Agar
ITS: Internal Transcribe Spacer
OTUs: Operational Taxonomic Units
IN: isolate number
ST: strain types
NCBI: National Center for Biotechnology Information
