# Supplementary figures and images for: The culturable endophytic fungal communities of switchgrass grown on a coal-mining site and their effects on plant growth
Source: PLoS One. 2018 Jun 14;13(6):e0198994. doi: 10.1371/journal.pone.0198994 (PMC6002093; doi:10.1371/journal.pone.0198994)

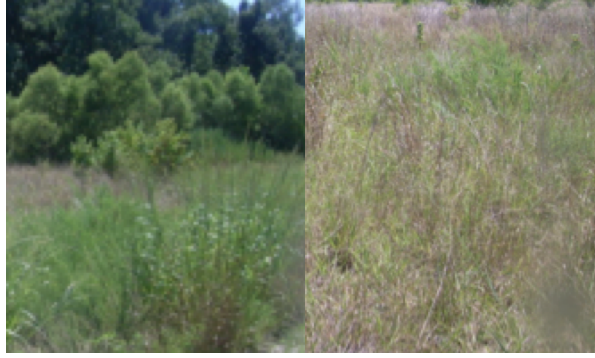

(A) Part 1

(B) Part 2

S1 Fig

Supplement: S1 Fig — (A) Swtichgrass grown on part1 with better vigorous growth. (B) Swtichgrass grown on part 2 with poor growth performance. (PDF) [file pone.0198994.s001.pdf]

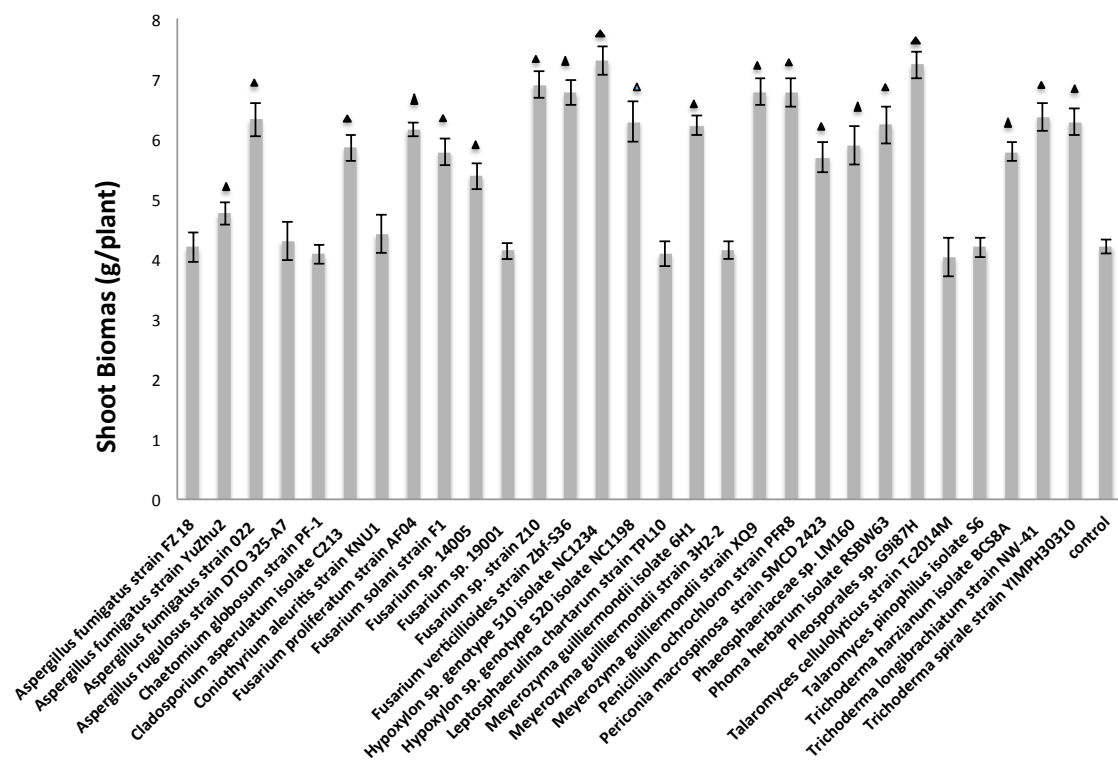

S2 Fig

Supplement: S2 Fig — The switchgrass plants were treated with water control and the water broth containing diverse fungal spores and mycelia at 8 weeks under greenhouse condition. The triangle represents the significant difference existing between the fungal broth treated and water treated plants and the data were further analyzed by the Student t-test (P<0.05). (PDF) [file pone.0198994.s002.pdf]
