# Supplementary material for: The culturable endophytic fungal communities of switchgrass grown on a coal-mining site and their effects on plant growth
Source: PLoS One. 2018 Jun 14;13(6):e0198994. doi: 10.1371/journal.pone.0198994 (PMC6002093; doi:10.1371/journal.pone.0198994)
Supplement: S1 Table — (PDF) [file pone.0198994.s003.pdf]

S1 Table

Class type (4) and isolate number	Order type (7) and isolate number	OTUs (22) at species level and isolate number
**Dothideomycetes (266)**	Capnodiales (22), Pleosporales (244)	*Cladosporium asperulatum (22), Coniothyrium aleuritis(23), Leptosphaerulina chartarum (19), Periconia macrospinosa (78), Phaeosphaeriaceae sp. (36), Phoma herbarum (23), Pleosporales sp. (65),*
**Eurotiomycetes (257)**	Eurotiales (257)	*Aspergillus fumigatus (83), Aspergillus rugulosus (25), Penicillium ochrochloron (89), Talaromyces cellulolyticus (38), Talaromyces pinophilus (22)*
**Saccharomycetes (175)**	Saccharomycetales (175)	*Meyerozyma guilliermondii (175)*
**Sordariomycetes (641)**	Sordariales (18), Hypocreales (457), Xylariales (166)	*Chaetomium globosum (18), Fusarium solani (18), Fusarium proliferatum (88), Fusarium sp (181), Fusarium verticillioides (22), Trichoderma harzianum (42), Trichoderma longibrachiatum (38), Trichoderma spiralen(68), Hypoxylon sp (166).*
