# Supplementary material for: The culturable endophytic fungal communities of switchgrass grown on a coal-mining site and their effects on plant growth
Source: PLoS One. 2018 Jun 14;13(6):e0198994. doi: 10.1371/journal.pone.0198994 (PMC6002093; doi:10.1371/journal.pone.0198994)
Supplement: S2 Table — The switchgrass plants were grown under greenhouse conditions. (PDF) [file pone.0198994.s004.pdf]

S2 Table

	Treatment (Fungal ID or Water)	Order	Height Average (CM)	SD (CM)	P Value (T-test)
1	***GQ923961.1 Pleosporales sp. G9i87H***	Pleosporales	98.29	5.90	<0.05
2	***JQ761870.1 Hypoxylon sp. genotype 510 isolate NC1234***	Xylariales	91.50	3.09	<0.05
3	***MF973465.1 Fusarium sp. strain Z10***	Hypocreales	89.75	3.36	<0.05
4	***KX065027.1 Fusarium verticillioides strain Zbf-S36***	Hypocreales	87.67	6.51	<0.05
5	***KU216711.1 Meyerozyma guilliermondii strain XQ9***	Saccharomycetales	87.00	3.24	<0.05
6	***KU321565.1 Aspergillus fumigatus strain 022***	Eurotiales	84.67	3.92	<0.05
7	***JQ761839.1 Hypoxylon sp. genotype 520 isolate NC1198***	Xylariales	83.08	3.10	<0.05
8	***KF619546 Meyerozyma guilliermondii isolate 6H1***	Saccharomycetales	81.83	3.27	<0.05
9	***MF187623.1 Fusarium solani strain F1***	Hypocreales	81.25	2.86	<0.05
10	***KU710251.1 Phoma herbarum isolate RSBW63***	Pleosporales	79.67	5.44	<0.05
11	***JQ658341.1 Periconia***	Pleosporales	77.25	2.98	<0.05

	macrospinosa strain SMCD 2423				
12	***KP686186.1 Trichoderma spirale strain YIMPH30310***	Hypocreales	74.83	4.84	<0.05
13	***LC168797.1 Cladosporium asperulatum isolate C213***	Capnodiales	72.67	2.63	<0.05
14	***MG596637.1 Trichoderma longibrachiatum strain NW-41***	Hypocreales	70.50	4.28	<0.05
15	***KJ921603.1 Coniothyrium aleuritis strain KNU1***	Pleosporales	70.04	2.86	<0.05
16	***KF358720.1 Penicillium ochrochloron strain PFR8***	Eurotiales	69.52	2.50	<0.05
17	***GU138648.1 Chaetomium globosum strain PF-1***	Sordariales	68.58	2.23	<0.05
18	***KU512836.1 Aspergillus fumigatus strain YuZhu2***	Eurotiales	67.75	4.10	<0.05
19	***EF060518.1 Phaeosphaeriaceae sp. LM160***	Pleosporales	67.33	2.05	<0.05
20	***MF426031.1 Fusarium proliferatum strain AF04***	Hypocreales	66.83	3.57	<0.05
21	***MF683087.1 Talaromyces pinophilus isolate S6***	Eurotiales	65.67	3.10	<0.05
22	***KU866664.1 Aspergillus rugulosus strain DTO 325-A7***	Eurotiales	65.00	3.43	<0.05

**23**	***KX258468.1 Meyerozyma guilliermondii strain 3H2-2***	Saccharomycetales	64.83	4.41	<0.05
**24**	***KP670444.1 Talaromyces cellulolyticus strain Tc2014M***	Eurotiales	64.50	2.81	0.23
**25**	***KP133165.1 Trichoderma harzianum isolate BCS8A***	Hypocreales	64.32	4.18	<0.05
**26**	***KJ863505.1 Leptosphaerulina chartarum strain TPL10***	Pleosporales	64.00	7.45	<0.05
**27**	***EU710827.1 Fusarium sp. 19001***	Hypocreales	62.06	6.50	0.11
**28**	***EU710819.1 Fusarium sp. 14005***	Saccharomycetales	60.75	3.96	0.22
**29**	***KX066060.1 Aspergillus fumigatus strain FZ 18***	Eurotiales	58.00	3.48	0.88
**Mock**	**Water**	N/A	58.80	5.52	N/A
