# Supplementary material for: The culturable endophytic fungal communities of switchgrass grown on a coal-mining site and their effects on plant growth
Source: PLoS One. 2018 Jun 14;13(6):e0198994. doi: 10.1371/journal.pone.0198994 (PMC6002093; doi:10.1371/journal.pone.0198994)
Supplement: S3 Table — (PDF) [file pone.0198994.s005.pdf]

S3 Table

1. GQ923961.1 *Pleosporales sp*. G9i87H

GCTCGGGGGAGCCCGTCGCTCGCGACGACGCTGCCTTGGGCGCTTAGCCCTTGACTATCA
CCTTGACTACGTGCACCTTTTGTTGTTTCCTCGGCAGGTCCTCTGCCGCCAGGAACCCCCC
AAACCCTTTTGCAACAGCATCCAAACTTCTGAAAACAAACCAAATCATTTACAACTTTTA
ACAATGGATCTCTTGGTTCTGGCATCGATGAAGAACGCAGCGAAATGCGATAAGTAGTG
TGAATTGCAGAATTCAGTGAATCATCGAATCTTTGAACGCACATTGCGCCCCATGGTATT
CCGTGGGGCATGCCTGTTCGAGCGTCATTTACCCCCTCAAGCTCCGCTTGGTGTTGGGCG
TCTGTCCCGCTTCGCGCGCGGACTCGCCCCAAAGGTATTGGCAGCGGTCGTGCCAGCTTC
TCGCGCAGCACATTGCGCTTCTCGAGGCACCGGTGGGCCCGCGTCCATCAAGCTCACCCCC
CCAGTTTGACCTCGGATCAGGTAGGGATACCCGCTGAACTTAAGCATATCAATAAGCGGA
GGCG

2. JQ761870.1 *Hypoxylon sp. genotype 510 isolate* NC1234

TTTGTGACCTTACTGCCAGGTGCCTCGGCGTGAGCTACGGCTACCCTGTAGCTACCCTAT
AGCTACCCTGCAGCTACCCTATAGTTGACCAGTAGCTACCCTGTAGTTACCCTATAATTA
CCCTGCAGCTACCCTATAGTTGACCAGTAGCTACCCTGTAGCCGGCTTATGGCCCGCCGA
AGGACCGCTAAACTCTTGTTTTTTACCACTGTTTCTCTGAATTTTAAACTTAAATAAGTT
AAAACTTTCAACAACGGATCTCTTGGTTCTGGCATCGATGAAAAACGCANCGAAATGCG
ATAAGTAATGTGAATTGCAGAATTCAATGAATCATCGAATCTTTGAACGCACATTGCGC
CCATTATTATTCTAGTGGGCATGCCTATTCNAGCGTCATTTCGACCCTTAAGCCCCTGTT
GCTTANCGTTGGGAATCTACAGCGTACTTCCTTAAAGTTAGTGGCGCAGTTAGGGTACA
CTCTCAGCGTAGTAATCTTTCTCGCGCGTGTGGTGGCCCTGGCTGCCAGCCGTTAAACCC
CTATATTTTATACGGATTGACCTCGTATTAG

3. MF973465.1 *Fusarium* sp. strain Z10

CCTCGCCGCGTGTACTGGCTCGGCCGGGCCTTTCCCTCTGTGGAACCCCATGCCCTTCACT
GGGCGTGGCGGGGAAACAGGACTTTTACTGTGAAAAAATTAGAGTGCTCCAGGCAGGCC
TATGCTCGAATACATTAGCATGGAATAATAGAATAGGACGTGTGGTTCTATTTTGTTGG
TTTCTAGGACCGCCGTAATGATTAATAGGGACAGTCGGGGGCATCAGTATTCAATTGTC
AGAGGTGAAATTCTTGGATTTATTGAAGACTAACTACTGCGAAAGCATTTGCCAAGGAT
GTTTTCATTAATCAGGAACGAAAGTTAGGGGATCGAAGACGATCAGATACCGTCGTAGT
CTTAACCATAAACTATGCCGACTAGGGATCGGACGGTGTTATTTTTTGACCCGTTCGGCA
CCTTACGAGAAATCAAAGTGCTTGGGCTCCAGGGGGAGTATGGTCGCAAGGCTGAAACT
TAAAGAAATTGACGGAAGGGCACCACCAGGGGTGGAGCCTGCGGCTTAATTTGACTCAA
CACGGGGAAACTCACCAGGTCCAGACACAATGAGGATTGACAGATTGAGAGCTCTTTCTT
GATTTTGTGGGTGGTGGTGCATGGCCGTTCTTAGTTGGTGGAGTGATTTGTCTGCTTAAT
TGCGATAACGAACGAGACCTTAACCTGCTAAATAGCCCGTATTGCTT

4. KX065027.1 *Fusarium verticillioides* strain Zbf-S36

ACCCCTGTGACTACCAATTGTTGCCTCGGCGGATCAGCCCGCTCCCGGTAAAACGGGACG
GCCCGCCAGAGGACCCCTAAACTCTGTTTCTATATGTAACTTCTGAGTAAAACCATAAAT
AAATCAAAACTTTCAACAACGGATCTCTTGGTTCTGGCATCGATGAATAACGCAGCAAA
ATGCGATAAGTAATGTGAATTGCAGAATTCAGTGAATCATCGAATCTTTGAACGCACAT
TGCGCCCGCCAGTATTCTGGCGGGCATGCCTGTTCGAGCGTCATTTCACCCCTCAAGCCCC
CGGGTTTGGTGTTGGGGATCGGNGAGCCCTTGGGGCAAACCGGCGCCGCCCGAATTGCGG
GGGCTCGTTGAT

5. KU216711.1 *Meyerozyma guilliermondii* strain XQ9

CGCTTACTGCGCGGCGAAAACCTTACACACAGTGTCTTTTTGATACAGAACTCTTGCTTT
GGTTTGGCCTAGAGATAGGTTGGGCCAGAGGTTTAACAAAACACAATTTAATTATTTTT
ACAGTTAGTCAAATTTTGAATTAATCTTCAAAACTTTCAACAACGGATCTCTTGGTTCTC
GCATCGATGAAGAACGCAGCGAAATGCGATAAGTAATATGAATTGCAGATTTTCGTGAA
TCATCGAATCTTTGAACGCACATTGCGCCCTCTGGTATTCCAGAGGGCATGCCTGTTTGA
GCGTCATTTCTCTCTCAAACCCCCGGGTTTGGTATTGAGTGATACTCTTAGTCGGACTAG
GCGTTTGCTTGAAAAGTATTGGCATGGGTAGTACTAGATAGTGCTGTCGACCTCTCAATG
TATTAGGTTTATCCAACTCGTTGAATGGTGTGGCGGGATATTTCTGGTATTGTTGGCCCG
GCCTTACAACAACCAAACAAGTTTGACCTCAAATCAGGTAGGAATACCCGCTGAACTTAA
GCATATCAATAAGCGGACGCTTGAATCATTACCGTATTCTTTTGCCAGCGCTTAAATGGC
CGGCGAAAAACCGTTACCACAGCGTCTTTTTGATACAGAAATCTGGCTTTGGTTTGGCCT
ATATATAGGNTTGGGNCACACGTTTAACAACCTTAATTTAATTAGTTTTTCGCTCCTCA
ATTTTTAATTAATCCTCACCATTCACACGAACTTTGCCCTCC

6. KU321565.1 *Aspergillus fumigatus* strain 022

CTGGGTCCACCTCCCACCCGTGTCTATCGTACCTTGTTGCTTCGGCGGGCCCGCCGTTTCG
ACGGCCGCCGGGGAGGCCTTGCGCCCCCGGGCCCGCGCCCGCCGAAGACCCCAACATGAAC
GCTGTTCTGAAAGTATGCAGTCTGAGTTGATTATCGTAATCAGTTAAAACTTTCAACAA
CGGATCTCTTGGTTCCGGCATCGATGAAGAACGCAGCGAAATGCGATAAGTAATGTGAA
TTGCAGAATTCAGTGAATCATCGAGTCTTTGAACGCACATTGCGCCCCCTGGTATTCCGG
GGGGCATGCCTGTCCGAGCGTCATTGCTGCCCTCAAGCACGGCTTGTGTGTTGGGCCCCC
GTCCCCCTCTCCCGGGGGACGGGCCCGAAAGGCAGCGGCGGCACCGCGTCCGGTCCTCGA
GCGTATGGGGCTTTGTCACCTGCTCTGTAGGCCCGGCCGGCGCCAGCCGACACCCAACTT
TATTTTTCTAAGGTTGACCTCGGATCANGTAGGGATACCCGCTGAACTTAAGCATATCA
ATA

7. JQ761839.1 *Hypoxylon sp.* genotype 520 isolate NC1198

TTGTGACCTTACTGTCGTTGCCTCGGCGTGAGCTACGGCTACCCTGTAGCTACCCTATAG
CTACCCTGCAGCTACCCTATAGTTGACCAGTAGCTACCCTGTAGTTACCCTATAGTTACC
CTGCAGCTACCCTATAGTTGACCAGTAGCTACCCTGTAGCCGGCTTATGGCCCGCCGAAG

GACCGCTAAACTCTTGTTTTTTACCACTGTTTCTCTGAATTTTAAACTTAAATAAGTTAA
AACTTTCAACAACGGATCTCTTGGTTCTGGCATCGATGAAGAACGCAGCGAAATGCGAT
AAGTAATGTGAATTGCAGAATTCAGTGAATCATCGAATCTTTGAACGCACATTGCGCCC
ATTAGTATTCTAGTGGGCATGCCTATTCGAGCGTCATTTCGACCCTTAAGCCCCTGTTGC
TTAGCGTTGGGAATCTACAGCGTAGTTCCTTAAAGTTAGTGGCGGAGTTAGGGTACACT
CTCAGCGTAGTAATCTTTCTCGCTCGTGTGGTGGCCCTGGCTGCTAGCCGTTAAACCCCT
ATATTTCTAGTGGTTGACCTCGGATTAGGTAGGAATACCCGCTGAACTTAAGCATATCA
ATAAGCGGAG

8. KF619546 *Meyerozyma guilliermondii* isolate 6H1

CNGCGCTTCCTGCGCGGCGAAAAACCTTACACACAGTGTCTTTTTGATACAGAACTCTTG
CTTTGGTTTGGCCTAGAGATAGGTTGGGCCAGAGGTTTAACAAAACACAATTTAATTAT
TTTTACAGTTAGTCAAATTTTGAATTAATCTTCAAAACTTTCAACAACGGATCTCTTGGT
TCTCGCATCGATGAAGAACGCAGCGAAATGCGATAAGTAATATGAATTGCAGATTTTCG
TGAATCATCGAATCTTTGAACGCACATTGCGCCCTCTGGTATTCCAGAGGGCATGCCTGT
TTGAGCGTCATTTCTCTCTCAAACCCCCGGGTTTGGTATTGAGTGATACTCTTAGTCGGA
CTAGGCGTTTGCTTGAAAAGTATTGGCATGGGTAGTACTAGATAGTGCTGTCGACCTCTC
AATGTATTAGGTTTATCCAACTCGTTGAATGGTGTGGCGGGATATTTCTGGTATTGTTG
GCCCGGCCTTACAACAACCAAACAAGTTTGACCTCAAATCAGGTAGGAATACCCGCTGAA
CTTAAGCATATCAATAAGCGGAGGAAGGAAGCGTACAGTATTCTTTTGCCAGCGCTTAA
TTGGCCGGGAAAAATCTT

9. MF187623.*1* *Fusarium solani* strain F11

CGCCTCNCCGCGTGTACTGGTCCGGCCGGGCCTTTCCCTCTGTGGAACCCCATGCCCTTCA
CTGGGTGTGGCGGGGAAACAGGACTTTTACTGTGAAAAAATTAGAGTGCTCCAGGCAGG
CCTATGCTCGAATACATTAGCATGGAATAATAGAATAGGACGTGTGGTTCTATTTTGTT
GGTTTCTAGGACCGCCGTAATGATTAATAGGGACAGTCGGGGGCATCAGTATTCAATTG
TCAGAGGTGAAATTCTTGGATTTATTGAAGACTAACTACTGCGAAAGCATTTGCCAAGG
ATGTTTTCATTAATCAGGAACGAAAGTTAGGGGATCGAAGACGATCAGATACCGTCGTA
GTCTTAACCATAAACTATGCCGACTAGGGATCGGACGGTGTTATATTTTGACCCGTTCGG
CACCTTACGAGAAATCAAAGTGCTTGGGCTCCAGGGGGAGTATGGTCGCAAGGCTGAAA
CTTAAAGAAATTGACGGAAGGGCACCACCAGGGGTGGAGCCTGCGGCTTAATTTGACTC
AACACGGGGAAACTCACCAGGTCCAGACACAATGAGGATTGACAGATTGAGAGCTCTTT
CTTGATTTTGTGGGTGGTGGTGCATGGCCGTTCTTAGTTGGTGGAGTGATTTGTCTGCTT
AATTGCGATAACGAACGAGACCTTAACCTGCTAAATAGCCCGTATTGCTTTGGCAGTACG
CTGGCTTCTTAGAGGGACTATCGGCTCAAGCCGATGGAAGTTTGAGGCAATAACAGGTCT
GTGATGCCCTTAGATGTTCTGGGCCGCACGCGCGCTACACTGACGGAGCCAGCGAGTACT
TCCTTGGCCGAAAGGCCCGGGTAATCTTGTTAAACTCCGTCGT

10. KU710251.1 *Phoma herbarum* isolate RSBW63

CGCCCTGCTCGCTCTTACCCATGTCTTTTGAGTACCTTACGTTTCCTCGGTGGGTTCGCC
CACCGATTGGACAAATTTAAACCCTTTGCAGTTGAAATCAGCGTCTGAAAAACTTTAAT
AATTACAACTTTCAACAACGGATCTCTTGGTTCTGGCATCGATGAAGAACGCAACGAAA
TGCGATAAGTATTGTGAATTGCANAATTCAGTGAATCATCGAATCTTTGAACGCACATT
GCGCCCCTTGGTATTCCATGGGGCATGCCTGTTCGAGCGTCATTTGTACCTCCAATCTCT
GCTTGGTGTAGGGTGTTTGTCTCGCGCAAAGCCCCATCAACTCCATGGCATCCCGCATTA
TGCTTTCCGACGTTATTAATTC

11. JQ658341.1 *Periconia macrospinosa* strain SMCD 2423

TCCGCGCTCCTTATACACCCACCCTCTGCCTACGTGTACCTCTATAGCTTCCTCGGCGGGC
TCGCCCGCCGCCAGGAACCCACGAAACCCCTTGCATTATACGCGAAAACTTCTGATAACA
AACCTAAATTATCACAACTTTCAACAATGGATCTCTTGGTTCTGGCATCGATGAAGAACG
CAGCGAAATGCGATAAGTAGTGTGAATTGCAGAATTCAGTGAATCATCGAATCTTTGAA
CGCACATTGCGGCCATAGGTATTCCTTTGGCCATGCCTGTTCGAGCGTCATTTACACCCT
CAAGCCTAGCTTGGTGTTGGGCGTCTGTCCCGCCGTTCTCGCGCGCGGACTCGCCTCAAA
GTCATTGGCGGCGGTCGTGCCGGCCCCCTCGCGCAGCACATTTGCGCTTCTCGGAGGCCCG
GCGGATCCGCGCTCCAGCAAGACCTTTCACGACTTGACCTCGGATCAGGTAGGGATACCC
GCTGAACTTAAGCATATCAATAAGCGAAGGAAT

12. KP686186.1 *Trichoderma spirale* strain YIMPH30310

TGTGACGTTACCAAACTGTTGCCTCGGCGGGATCTCTGCCCCGGGTGCGTCNCAGCCCCG
GACCAAAGCGCCCGCCTGAGGACCAACCAAAACTCTTTTGTATACCCCCTCGCGGGTTTT
TATATCTGANCCATCTCGGCGCCTCTCNTAGGCGTTTCGAAAATGAATCAAAACTTTCAT
CAGCGGATCTCTTGGTTCTGGCNACNATGACGAACCCTCCTAAATGTGATAAGTAAGGG
GATTTGCNGAATTCAGNGATTCATCTTATCTTTNAACTCTCATTGCGCCCGCGACCAGCC
TGGCGGGACTGTGTGTCAGAGCGTCATTTCGCCCCTCAACCCCGGAGGGGGGTC

13. LC168797.1 *Cladosporium asperulatum* isolate C213

CMCCGGGATGTTCATAACCCTTTGTTGTCCGACTCTGTTGCCTCCGGGGCGACCCTGCCT
TCGGGCGGGGGCTCCGGGTGGACACTTCAAACTCTTGCGTAACTTTGCAGTCTGAGTAAA
CTTAATTAATAAATTAAAACTTTTAACAACGGATCTCTTGGTTCTGGCATCGATGAAGA
ACGCAGCGAAATGCGATAAGTAATGTGAATTGCAGAATTCAGTGAATCATCGAATCTTT
GAACGCACATTGCGCCCCCTGGTATTCCGGGGGGCATGCCTGTTCGAGCGTCATTTCACC
ACTCAAGCCTCGCTTGGTATTGGGCAACGCGGTCCGCCGCGTGCCTCAAATCGACCGGCT
GGGTCTTCTGTCCCCTAAGCGTTGTGGAAACTATTCGCTAAAGGGTGTTCGGGAGGCTAC
GCCGTAAAACAACCCCATTTCTAAGGTTGACCTCGGATCAGGTAGGGATACCCGCTGAAC
TTAAGCATATCAATAAGCGGAGGAA

14. MG596637.1 *Trichoderma longibrachiatum* strain NW-41

AGGAGGGCCGGGTGCGTGGGCGCCCGCCTGTAGACAAAGGGGGGAGGCGTGTGTGCGCGC
GTTCACATCTCCAGTCGCGGCTCGGTTTTATTTTTGCTCTGATCCTTTCTCGGCGACCCG
AGGGGGGGGCGTGAAGATGAATCAAAACTTTCAACAACGGATCTCTTGGTTCTGGCATC
GATGAAGAACGCAGCGAAATGCGATAAGTAATGTGAATTGCACAATTCAGTGAATCATC
GAATCTTTGAACGCACATTGCGCCCGCCAGTATTCTGGCGGGCATGCCTGTCCGAGCGTC
ATTTCAACCCTCNAACCCCTCCGGGGGGTCGGCGTTGGGGATCGGCCCCTCACCGGGCCG
CCCCCGAAATACAGTGGCGGTCTCGCCGCAGCCTCTCCTGCGCAGTAGTTTGCACACTCN
CACCGGGAGCGCGGCGCGGCCACAGCCGTAAAACACCCCAAACTTCTGAAATGTTGACCT
CGGATCAGGTAGGAATACCCGCTGAACTTAAGCATATCAATAANCGGAGGAACGCTNCG
TGAGTCTGCCTCTCTTTACGCACGGGGAAAGG

15. KJ921603.1 *Coniothyrium aleuritis* strain KNU1

TGCTATCTCTTACCCATGTCTTTTGAGTACCTTACGTTTCCTCGGTGGGTTCGCCCACCG
ATTGGACAAATTTAAACCCTTTGCAGTTGAAATCAGCGTCTGAAAAAACTTAATAGTTA
CAACTTTCAACAACGGATCTCTTGGTTCTGGCATCGATGAAGAACGCAGCGAAATGCGAT
AAGTAGTGTGAATTGCAGAATTCAGTGAATCATCGAATCTTTGAACGCACATTGCGCCCC
TTGGTATTCCATGGGGCATGCCTGTTCGAGCGTCATTTGTACCTTCAAGCTCTGCTTGGT
GTTGGGTGTTTGTCTCCTGTAGACTCGCCTTAAAACAATTGGCAGCCGGCGTATTGATTT
CGGAGCGCAGTACATCTCGCGCTTTGCACTCATAACGACGACATCCAAAAGTACATTTTT
ACACTCTTGACCTCGGATCAGGTAGGGATACCCGCTGAACTTAAGCATATCAATAAGCGG
AGGANNAANNNTTACCTANAGTTGTAGGCTTTGCCTGCTATCTCTTACCCATGTCTTTT
GAGTACCTTACGTTTCCTCNGTGGGTTCGCCCACCGATTGGACAAATTTANACCCTTTGC
AGTTGAAANTCA

16. KF358720.1 *Penicillium ochrochloron* strain PFR8

ACCTCCCACCCGTGTTTATCGTACCTTGTTGCTTCGGCGGGCCCGCCTCACGGCCGCCGGG
GGGCATCCGCCCCCGGGCCCGCGCCCGCCGAAGACACCATTGAACTCTGTCTGAAGATTG
CAGTCTGAGCGATTAGCTAAATCAGTTAAAACTTTCAACAACGGATCTCTTGGTTCCGGC
ATCGATGAAGAACGCAGCGAAATGCGATACGTAATGTGAATTGCAGAATTCAGTGAATC
ATCGAGTCTTTGAACGCACATTGCGCCCCCTGGTATTCCGGGGGGCATGCCTGTCCGAGC
GTCATTGCTGCCCTCAAGCACGGCTTGTGTGTTGGGCCCCGCCCCCCGGTTCCGGGGGGC
GGGCCCGAAAGGCAGCGGCGGCACCGCGTCCGGTCCTCGAGCGTATGGGGCTTTGTCACC
CGCTCTGTAGGCCCGGCCGGCGCCCGCCGGCGACCCCAAATCAATCTATCCAGGTTGACCT
CGGATCAGGTAGGGATACCCGCTGAACTTAAGCATATCAATAAGCGGA

17. GU138648.1 ***Chaetomium globosum*** **strain PF-1**

ACTCCCGGACCGTTGTGACGTTACCTATACCGTTGCTTCGGCGGGCGGCCCCGGGGTTTA
CCCCCCGGGCGCCCCTGGGCCCCACCGCGGGCGCCCGCCGGAGGTCACCAAACTCTTGATA
ATTTATGGCCTCTCTGAGTCTTCTGTACTGAATAAGTCAAAACTTTCAACAACGGATCTC
TTGGTTCTGGCATCGATGAAGAACGCAGCGAAATGCGATAAGTAATGTGAATTGCAGAA
TTCAGTGAATCATCGAATCTTTGAACGCACATTGCGCCCGCCAGCATTCTGGCGGGCATG
CCTGTTCGAGCGTCATTTCAACCATCAAGCCCCCGGGCTTGTGTTGGGGACCTGCGGCTG
CCGCAGGCCCTGAAAAGCAGTGGCGGGCTCGCTGTCGCACCGAGCGTAGTAGCATACATC
TCGCTCTGGTCGCGCCGCGGGTTCCGGCCGTTAAACCACCTTTTAACCCAAGGTTGACCT
CGGATCAGGTAGGAAGACCCGCTGAACTTAAGCATATCAATACCGGATCGGAA

18. KU512836.1 ***Aspergillus fumigatus*** **strain YuZhu2**

GNCGCCTCNCCGCGAGTACTGGTCCGGCTGGACCTTTCCTTCTGGGGAACCTCATGGCCT
TCACTGGCTGTGGGGGGAACCAGGACTTTTACTGTGAAAAAATTAGAGTGTTCAAAGCA
GGCCTTTGCTCGAATACATTAGCATGGAATAATAGAATAGGACGTGCGGTTCTATTTTG
TTGGTTTCTAGGACCGCCGTAATGATTAATAGGGATAGTCGGGGGCGTCAGTATTCAGC
TGTCAGAGGTGAAATTCTTGGATTTGCTGAAGACTAACTACTGCGAAAGCATTCGCCAA
GGATGTTTTCATTAATCAGGGAACGAAAGTTAGGGGATCGAAGACGATCAGATACCGTC
GTAGTCTTAACCATAAACTATGCCGACTAGGGATCGGGCGGTGTTTCTATGATGACCCGC
TCGGCACCTTACGAGAAATCAAAGTTTTTGGGTTCTGGGGGGAGTATGGTCGCAAGGCT
GAAACTTAAAGAAATTGACGGAAGGGCACCACAAGGCGTGGAGCCTGCGGCTTAATTTG
ACTCAACACGGGGAAACTCACCAGGTCCAGACAAAATAAGGATTGACAGATTGAGAGCT
CTTTCTTGATCTTTTGGATGGTGGTGCATGGCCGTTCTTAGTTGGTGGAGTGATTTGTCT
GCTTAATTGCGATAACGAACGAGACCTCGGCCCTTAAATAGCCCGGTCCGCATTTGCGGG
CCGCTGGCTTCTTANGGGGACTATCGGCTCAAGCCGATGGAAGTGCGCGGCAATAACAGG
TCTGTGATGCCCTTAGATGTTCTGGGCCGCACGCGCGCTACACTGACAGGGNCAGCGAGT
ACATCACCTTGGCCGAGAGGTCTGGGTAATCTTGTTAAACCCTGTCGTGCTGGGGATAGA
GCATTGCAATTATTGCTCTTCAACGAGGAATGNCTANTAGGCACGAGTCATCAGCTCGN
GCCGATTACGTCCCTGCCCTTTGNACACACCGCCCGTCGCTACTACCGATTGAATGGCTC
G

19. EF060518.1 ***Phaeosphaeriaceae*** **sp. LM160**

GTAGCAATACAGCCCAAAGGCCTCTTCTATTACCCTTGTTTTTGAGTACCTATGTTTCCT
TGGTGGGCTTGCCTGCCAATAGGACAAACTATTAAACCTTTTTTAATTTTCAATCAGCGT
CTGAATAAACTTTAATAATTACAACTTTCAACAACGGATCTCTTGGTTCTGGCATCGATG
AAGAACGCAGCGAAATGCGATAAGTAGTGTGAATTGCAGAATTCAGTGAATCATCGAATC
TTTGAACGCACATTGCGCCCCTTGGTATTCCATGGGGCATGCCTGTTCGAGCGTCATTTGT
ACCTTCAAGCTCTGCTTGGTGTTGGGTGTTTTGTCCTCTCCATTGCGTTTGGACTCGCCTT
AAAGCAATTGGCAGCCAGTGTATTGGTTTTAAGCGCAGCACATCTTGCGTCTTTTTCCTA
TAACACTCGCGTCCATTAAGCCTTTTTATCACTTTTGACCTCGGATCAGGTAGGGATACCC
GCTGAACTTAAGCATATCAATAAGCGGAGGAANGAACCGGCACATTCNCGCCCCGGGTGC

GTCCATCCACCCCCGCCAACTCCTGACACGTTTGGTTCCAGATAAAGTGTGTCTAGGGGGA
GAGTGGC

20. MF426031.1 *Fusarium proliferatum* strain AF04

ACCCCTGTGACTACCAATTGTTGCCTCGGCGGATCAGCCCGCTCCCGGTAAAACGGGACG
GCCCGCCAGAGGACCCCTAAACTCTGTTTCTATATGTAACTTCTGAGTAAAACCATAAAT
AAATCAAAACTTTCAACAACGGATCTCTTGGTTCTGGCATCGATGAATAACGCAGCAAA
ATGCGATAAGTAATGTGAATTGCAGAATTCAGTGAATCATCGAATCTTTGAACGCACAT
TGCGCCCGCCAGTATTCTGGCGGGCATGCCTGTTCGAGCGTCATTTCACCCCTCAAGCCCC
CGGGTTTGGTGTTGGGGATCGGNGAGCCCTTGGGGCAAACCGGCGCCGCCCGAATTGCGG
GGGCTCGTTGAT

21. MF683087.1 *Talaromyces pinophilus* isolate S6

GCACCTCCCACCCTTGTCTCTATACACCTGTTGCTTTGGCGGGCCCACCGGGGCCACCTGG
TCGCCGGGGGACGCACGTCCCCGGGCCCGCGCCCGCCGAAGCGCTCTGTGAACCCTGATG
AAGATGGGCTGTCTGAGTACTATGAAAATTGTCAAAACTTTCAACAATGGATCTCTTGG
TTCCGGCATCGATGAAGAACGCAGCGAAATGCGATAAGTAATGTGAATTGCAGAATTCC
GTGAATCATCGAATCTTTGAACGCACATTGCGCCCCCTGGCATTCCGGGGGGCATGCCTG
TCCGAGCGTCATTTCTGCCCTCAAGCACGGCTTGTGTGTTGGGTGTGGTCCCCCCGGGGA
CCTGCCCGAAAGGCAGCGGCGACGTCCGTCTGGTCCTCGAGCGTATGGGGCTCTGTCACT
CGCTCGGGAAGGACCTGCGGGGGTTGGTCACCACCATGTTTTACCACGGTTGACCTCGGA
TCAGGTAGGAGTTACCCGCTGAACTTAAGCATATCAARR

22. KU866664.1 *Aspergillus rugulosus* strain DTO 325-A7

AACACTGTTGCTTCGGCGGGGAGCCCCCCAGGGGCGAGCCGCCGGGGACCACTGAACTTC
ATGCCTGAGAGTGATGCAGTCTGAGCCTGAATACAAATCAGTCAAAACTTTCAACAATG
GATCTCTTGGTTCCGGCATCGATGAAGAACGCAGCGAACTGCGATAAGTAATGTGAATT
GCAGAATTCAGTGAATCATCGAGTCTTTGAACGCACATTGCGCCCCCTGGCATTCCGGGG
GGCATGCCTGTCCGAGCGTCATTGCTGCCCTCAAGCCCGGCTTGTGTGTTGGGTCGTCGT
CCCCCCCGGGGGACGGGCCCGAAAGGCAGCGGCGGCACCGTGTCCGGTCCTCGAGCGTAT
GGGGCTTTGTCACCCGCTCGATTAGGGCCGGCCGGGCGCCAGCCGGCGTCTCCAACCTTA
TTTTTCTCAGGTTGACCTCCGATCAGGTAGGGAAACCCGCTGAACTTTACCATATCACTA
AGGGGAAGAA

23. KX258468.1 *Meyerozyma guilliermondii* strain 3H2-2

ACCGAGCCTTTCCTTCTGGCTAACCATTCGCCCTTGTGGTGTTTGGCGAACCAGGACTTT
TACTTTGAAAAAATTAGAGTGTTCAAAGCAGGCCTTTGCTCGAATATATTAGCATGGAA
TAATAGAATAGGACGTTATGGTTCTATTTTGTTGGTTTCTAGGACCATCGTAATGATTA
ATAGGGACGGTCGGGGGCATCAGTATTCAGTTGTCAGAGGTGAAATTCTTAGATTTACT
GAAGACTAACTACTGCGAAAGCATTTGCCAAGGACGTTTTCATTAATCAAGAACGAAAG

TTAGGGGATCGAAGATGATCAGATACCGTCGTAGTCTTAACCATAAACTATGCCGACTA
GGGATCGGGTGTTGTTCTTTTTTTGACGCACTCGGCACCTTACGAGAAATCAAAGTCTTT
GGGTTCTGGGGGGAGTATGGTCGCAAGGCTGAAACTTAAAGGAATTGACGGAAGGGCAC
CACCAGGAGTGGAGCCTGCGGCTTAATTTGACTCAACACGGGGAAACTCACCAGGTCCAG
ACACAATAAGGATTGACAGATTGAGAGCTCTTTCTTGATTTTGTGGGTGGTGGTGCATG
GCCGTTCTTAGTTGGTGGAGTGATTTGTCTGCTTAATTGCGATAACGAACGAGACCTTAA
CCTACTAAATAGTGCTGCTAGCTTTTGCTGGTATAGTCACTTTCTTANAGGGACTATCGA
TTT

24. KP670444.1 *Talaromyces cellulolyticus* strain Tc2014M

GCGGCACCTCCCACCCTTGTCTCTATACACCTGTTGCTTTGGCGGGCCCACCGGGGTTATT
TGGTCGCCGGGGGACGCACGTCCCCGGGCCCGCGCCCGCCGAAGCGCTCTGTGAACCCTG
ATGAAGATGGGCTGTCTGAGTACTATGAAAATTGTCAAAACTTTCAACAATGGATCTCT
TGGTTCCGGCATCGATGAAGAACGCAGCGAAATGCGATAAGTAATGTGAATTGCAGAAT
TCCGTGAATCATCGAATCTTTGAACGCACATTGCGCCCCCTGGCATTCCGGGGGGCATGC
CTGTCCGAGCGTCATTTCTGCCCTCAAGCACGGCTTGTGTGTTGGGTGTGGTCCCCCCGG
GGACCTGCCCGAAAGGCAGCGGCGACGTCCGTCTGGTCCTCGAGCGTATGGGGCTCTGTC
ACTCGCTCGGGAAGGACCTGCGGGGGTTGGTCACCACCATATTTTACCACGGTTGACCTC
GGATCAGGTAGGAGTTACCCGCTGAACTTAAGCATATCAAA

25. KP133165.1 *Trichoderma harzianum* isolate BCS8A

GCGTGCACTGGTCCGGCCGGGCCTTTCCCTCTGCGGAACCCCATGCCCTTCACTGGGTGTG
GCGGGGAAACAGGACTTTTACTTTGAAAAAATTAGAGTGCTCAAGGCAGGCCTATGCTC
GAATACATTAGCATGGAATAATAGAATAGGACGTGTGGTTCTATTTTGTTGGTTTCTAG
GACCGCCGTAATGATTAATAGGGACAGTCGGGGGCATCAGTATTCAATTGTCAGAGGTG
AAATTCTTGGATTTATTGAAGACTAACTACTGCGAAAGCATTTGCCAAGGATGTTTTCA
TTAATCAGGAACGAAAGTTAGGGGATCGAAGACGATCAGATACCGTCGTAGTCTTAACC
ATAAACTATGCCGACTAGGGATCGGACGATGTTACATTTTTGACGCGTTCGGCACCTTAC
GAGAAATCAAAGTGCTTGGGCTCCAGGGGGAGTATGGTCGCAAGGCTGAAACTTAAAGA
AATTGACGGAAGGGCACCACCAGGGGTGGAGCCTGCGGCTTAATTTGACTCAACACGGGG
AAACTCACCAGGTCCAGACACAATGAGGATTGACAGATTGAGAGCTCTTTCTTGATTTTG
TGGGTGGTGGTGCATGGCCGTTCTTAGTTGGTGGAGTGATTTGTCTGCTTAATTGCGATA
ACGAACGAGACCTTAACCTGCTAAATAGCCCGTATTGCTTTGGCAGTACGCCGGCTTCTT
AGAGGGACTATCGGCTCAAGCCGATGGAAGTTTGANGCAATAACAGGTCTGTGATGCCC
TTAGATGTTCTGGGCCGCACGCGCGCTACACTGACGGANCCAGCGAGTACTCCCTTGGCC
GGG

26. KJ863505.1 *Leptosphaerulina chartarum* strainTPL10

GCGCGGCCCCCGAGGAGCGGAAACAATCCTTGGGAGGTATGCGGGGGCTTCGAGCCCCCC
ATTTACGCACGCACGACTGCCATCCTTACTTTACGAGCACCTTCTGTTCTCCCTCGGCGG
GGCAACCTGCCGTTGGAACCGAATAAACTCTTTTTGCATCTAGCATTACCTGTTCCGAAA

CAAACAATCGTTACAACTTTCAACAATGGATCTCTTGGCTCTGGCATCGATGAAGAACGC
AGCGAAATGCGATAAGTAGTGTGAATTGCAGAATTCAGTGAATCATCGAATCTTTGAAC
GCACATTGCGCCCCTCGGTATTCCGTGGGGCATGCCTGTTCGAGCGTCATCTACACCCTC
AAGCTCTGCTTGGTGTTGGGCGTCTGTCCCGCCTCCGCGCGTGGACTCGCCCCAAATTCA
TTGGCAGCGGTCCTTGCCTCCTCTCGCGCAGCACATTGCGCTTCTCGAGGGGCTACGGCTC
GCGTCCAACAAGCACATTTACCGTCTTTGACCTCGGATCAGGTAGGGATACCCGCTGAAC
TTAAGCATATCA

27. EU710827.*1* *Fusarium sp.* 19001

GCGTGTACTGGTCCGGCCGGGCCTTTCCCTCTGTGGAACCCCATGCCCTTCACTGGGTGT
GGCGGGGAAACAGGACTTTTACTGTGAAAAAATTAGAGTGCTCCAGGCAGGCCTATGCT
CGAATACATTAGCATGGAATAATAGAATAGGACGTGTGGTTCTATTTTGTTGGTTTCTA
GGACCGCCGTAATGATTAATAGGGACAGTCGGGGGCATCAGTATTCAATTGTCAGAGGT
GAAATTCTTGGATTTATTGAAGACTAACTACTGCGAAAGCATTTGCCAAGGATGTTTTC
ATTAATCAGGAACGAAAGTTAGGGGATCGAAGACGATCAGATACCGTCGTAGTCTTAAC
CATAAACTATGCCGACTAGGGATCGGACGGTGTTATATTTTGACCCGTTCGGCACCTTAC
GAGAAATCAAAGTGCTTGGGCTCCAGGGGGAGTATGGTCGCAAGGCTGAAACTTAAAGA
AATTGACGGAAGGGCACCACCAGGGGTGGAGCCTGCGGCTTAATTTGACTCAACACGGGG
AAACTCACCAGGTCCAGACACAATGAGGATTGACAGATTGAGAGCTCTTTCTTGATTTTG
TGGGTGGTGGTGCATGGCCGTTCTTAGTTGGTGGAGTGATTTGTCTGCTTAATTGCGATA
ACGAACGAGACCTTAACCTGCTAAATAGCCCGTATTGCTTTGGCAGTACGCTGGCTTCTT
AGAGGGACTATCGGCTCAAGCCGATGGAAGTTTGAGGCAATAACAGGTCTGTGATGCCC
TTAGATGTTCTGGGCCGCACGCGCGCTACACTGACGGAGCCAGCGAGTACTTCCTTGGCC
GAAAGGCCCGGGTAATCTTGTTAAACTCCGTCGTGCTGGGGATAGAGCATTGCAATTATT
GCTCTTCNACGAGGAATCCCTAGTAAGCGCAAGTCATCAGCTTGCGTTGATTACGTCCCT
GCCCTTTGTACACACNGCCCGTCGCTACTACCGATTGAATGGCTCA

28. EU710819.1 ***Fusarium sp*. 14005**

CGCCTCNCCGCGTGTACTGGTCCGGCCGGGCCTTTCCCTCTGTGGAACCCCATGCCCTTCA
CTGGGTGTGGCGGGGAAACAGGACTTTTACTGTGAAAAAATTAGAGTGCTCCAGGCAGG
CCTATGCTCGAATACATTAGCATGGAATAATAGAATAGGACGTGTGGTTCTATTTTGTT
GGTTTCTAGGACCGCCGTAATGATTAATAGGGACAGTCGGGGGCATCAGTATTCAATTG
TCAGAGGTGAAATTCTTGGATTTATTGAAGACTAACTACTGCGAAAGCATTTGCCAAGG
ATGTTTTCATTAATCAGGAACGAAAGTTAGGGGATCGAAGACGATCAGATACCGTCGTA
GTCTTAACCATAAACTATGCCGACTAGGGATCGGACGGTGTTATATTTTGACCCGTTCGG
CACCTTACGAGAAATCAAAGTGCTTGGGCTCCAGGGGGAGTATGGTCGCAAGGCTGAAA
CTTAAAGAAATTGACGGAAGGGCACCACCAGGGGTGGAGCCTGCGGCTTAATTTGACTC
AACACGGGGAAACTCACCAGGTCCAGACACAATGAGGATTGACAGATTGAGAGCTCTTT
CTTGATTTTGTGGGTGGTGGTGCATGGCCGTTCTTAGTTGGTGGAGTGATTTGTCTGCTT
AATTGCGATAACGAACGAGACCTTAACCTGCTAAATAGCCCGTATTGCTTTGGCAGTACG
CTGGCTTCTTAGAGGGACTATCGGCTCAAGCCGATGGAAGTTTGAGGCAATAACAGGTCT
GTGATGCCCTTAGATGTTCTGGGCCGCACGCGCGCTACACTGACGGAGCCAGCGAGTACT
TCCTTGGCCGAAAGGCCCGGGTAATCTTGTTAAACTCCGTCGTGCTGGGGATAGAGCATT

GCAATTATTGCTCTTCCACGAGGAATCCCTAGTAAGCGCAAGTCATCAGCTTGCGTTGAT
TACGTCCCTGCCCTTTGTACACACNGCCCGTCGCTACTACCGATTGAATGGCTCANTGAT
GCGTCCGNACTGGCCCCCGCCGGTGGGCATTACCGCTCTGGCGGGAAACTCTACC

29. KX066060.1 *Aspergillus fumigatus* strain FZ 18

GCCCCTGGGTCACCTCCCACCCGTGTCTATCGTACCTTGTTGCTTCGGCGGGCCCGCCGTT
TCGACGGCCGCCGGGGAGGCCCTGCGCCCCCGGGCCCGCGCCCGCCGAAGACCCCAACATG
AACGCTGTTCTGAAAGTATGCAGTCTGAGTTGATTATCGTAATCANTTAAAACTTTCAA
CAACGGATCTCTTGGTTCCGGCATCGATGAAGAACGCAGCGAAATGCGATAAGTAATGT
GAATTGCAGAATTCAGTGAATCATCGAGTCTTTGAACGCACATTGCGCCCCCTGGTATTC
CGGGGGGCATGCCTGTCCGAGCGTCATTGCTGCCCTCAAGCACGGCTTGTGTGTTGGGCC
CCCGTCCCCCTCTCCCGGGGGACGGGCCC
